# Impact of a multidisciplinary approach involving clinical pharmacist on resolving drug related problems in chronic kidney patients: a prospective interventional study

**DOI:** 10.1186/s12882-023-03210-5

**Published:** 2023-05-26

**Authors:** Aysel Pehlivanli, Sahin Eyupoglu, Bilgen Basgut, Sehsuvar Erturk, A. Tanju Ozcelikay

**Affiliations:** 1 Faculty of Pharmacy, Department of Clinical Pharmacy, Ankara University, Ankara, Turkey; 2 Faculty of Pharmacy, Department of Pharmacology, Baskent University, Ankara, Turkey; 3 School of Medicine, Department of Nephrology, Ankara University, Ankara, Turkey; 4 Faculty of Pharmacy, Department of Pharmacology, Ankara University, Ankara, Turkey

**Keywords:** Chronic kidney disease, Drug-related problems, Clinical pharmacist, PCNE

## Abstract

**Background:**

Chronic kidney disease (CKD) is a major public health concern due to its high mortality risk, high hospitalization rates and cost, and low life expectancy. Thus, CKD patients are among patient group that may benefit from clinical pharmacy services the most.

**Methods:**

This was a prospective interventional study conducted between October 1, 2019, and March 18, 2020, in the nephrology ward of Ankara University School of the Medicine, Ibn-i Sina Hospital. DRPs were classified according to PCNE v8.03. The main outcomes were interventions proposed and the rate of acceptance by the physicians.

**Results:**

269 pre-dialysis patients were included to determine DRPs during the treatment process of the patients. 205 DRPs were found in 131 (48.7%) patients. Treatment efficacy was found to be the main type of DRPs (56.2%) followed by treatment safety (39.6%). When patients with and without DRPs were compared, it was found that the number of female patients (55.0%) was higher in the group with DRPs (p < 0.05). The length of hospital stays (11.3 ± 7.7) and the mean number of drugs used (9.6 ± 3.6) in the group with DRPs were significantly higher than those without DRPs (9.3 ± 5.9; 8.1 ± 3.5, respectively) (p < 0.05). 91.7% of the interventions were accepted by the physicians, and patients and found clinically beneficial. 71.7% of DRPs were fully resolved, 1.9% partially resolved and 23.4% could not be resolved.

**Conclusions:**

A high prevalence of DRPs in patients with chronic kidney disease was determined during therapy. Clinical pharmacist interventions were highly accepted by the physicians and patients. This may indicate implementation of clinical pharmacy services in the nephrology ward has a great impact on optimized therapy and prevention DRPs.

## Introduction

Due to its high mortality risk, chronic kidney disease (CKD) is a significant health issue globally [1]. CKD is defined as abnormalities in kidney structure and function that last longer than three months, according to the KDIGO guidelines [2]. The prevalence of CKD in the world is estimated to be between 11% and 13% [3]. According to World Health Organization data from 2013, CKD is responsible for 1.5% of all deaths worldwide [4]. 15.7% of population in our country have CKD, according to the CREDIT survey carried out by the Turkish Society of Nephrology [5].

CKD is an important public health problem due to its high mortality risk and high hospitalization rates. [6]. It has been established that clinical pharmacy services are crucial for CKD patients who require a complicated course of treatment [7]. With the use of this service, medical professionals such as physicians, nurses, dietitians, and clinical pharmacists will work together as a multidisciplinary team to better manage CKD-related comorbid disorders and stop the course of the disease [7]. In a recently released meta-analysis study that solely looked at CKD patients who were hospitalized, the prevalence of DRP was found to be between 12 and 77% [8]. This meta-analysis provides evidence that DRPs are a frequent occurrence and burden for hospitalised patients with CKD [8].

The management and resolution of DRP in adult CKD patients have been shown to improve disease-oriented and patient-oriented outcomes, which include treatment management [9, 10], adherence and knowledge of treatment [11, 12], patient’s quality of life [13], hospitalisation rate and length of stay [14] and cost to the healthcare system [15].

This is the first study that reports DRPs in nephrology ward by clinical pharmacists in Turkey. Through the intervention of clinical pharmacists, potential medication errors and adverse drug reactions can be effectively avoided, and the medication safety of patients can be further.

### Aim of the study

The main goal of this study is to assess and resolve the drug-related problems in CKD patients who are hospitalized at the Nephrology Clinic of the Ankara University Faculty of Medicine considering the KDIGO classification. The study was approved by the Ethics Committee for Human Research of the Ankara University School of Medicine (Date: September 12, 2019; No: İ3-70-19).

## Methods

### Study design and setting

This is a prospective study conducted between October 1, 2019, and March 18, 2020, in the nephrology ward of Ankara University School of the Medicine, Ibn-i Sina Hospital. The hospital is a 1000-bed tertiary care hospital and 34 of them in Nephrology clinic which is staffed by 10 nephrologist and 17 nurses. Until this study, clinical pharmacy services were not provided in the Nephrology service.

### Sample size and Study Population

The number of patients (n) to be included in the sample of the study was calculated as at least 94 patients when calculated with a 95% confidence interval, 5% margin of error, and 80% frequency of drug-related problems [16] using the Raosoft® sample calculation program [17].

#### Inclusion criteria

All patients who were admitted to Ankara University School of Medicine Nephrology Clinic and met the following criteria,

- Eighteen years and older,
- Pre-dialysis patients (Stage 1–5 CKD) considering KDIGO classification,
- With or without comorbidity, were included in the study.

### Exclusion criteria

- Patients who received chronic dialysis treatment,
- Patients who did not give consent for the study,
- Patients with incomplete files or missing information were not included.

### Drug-related problems and Pharmaceutical Care Network Europe (PCNE)

PCNE; defines an event or situation that prevents the desired results from being achieved in treatment as a DRP. PCNE is a tool used to classify DRP. The PCNE consists of five main sections that report problems, causes, planned interventions, intervention acceptance, and the status of DRP. Problems are classified into three sections treatment effectiveness, safety, and other. In treatment efficacy, there is a problem with the (lack of) effect of pharmacotherapy. In treatment safety, an adverse drug event may have occurred in the patient. The reasons for DRP consist of 8 parts drug selection, drug form, dose selection, treatment duration, dispensing, drug use process, patient-related, and others. The planned intervention consists of 5 sections no intervention, at the prescriber level, at the patient level, at the drug level, and others. While intervention acceptance consists of 3 parts, intervention accepted, intervention not accepted, and others, the status of the DRP consists of 4 parts, problem status unknown, problem solved, problem partially solved, and problem not solved [18].

#### Pharmacist intervention

A clinical pharmacist participated in rounds with physicians during the study period, and laboratory results, drugs used, and non-drug products were assessed daily. Physicians and patients were advised and counselled to identify, prevent, or solve the problems related to the drugs (side effects, drug misuse, unnecessary drug use, drug interaction, drug use in missing or overdose, drug adverse events, and storage of drugs, etc.) used by patient with CKD. Recommendations were actively made during the ward round in real-time by the clinical pharmacist. Interventions for each identified DRP were discussed with the prescriber, and appropriate recommendations were suggested to resolve the problem. The clinical pharmacist used the latest guidelines (such as the KDIGO) and standardized databases such as the British National Formulary (BNF), Medscape, UpToDate, and Drugs.com for the prevention and resolution of DRPs. The clinical pharmacist evaluated the outcome of each recommendation, the nephrologist confirmed the results. PCNE V 8.03 was used to classify drug-related problems [18].

### Data Collection and statistical analysis

The data were collected from the patient’s medical records and patients and/or physicians’ interviews. Quantitative data were expressed as mean, standard deviation, median, maximum, and lowest values, percentages, and qualitative data were expressed as numbers and percentages in the statistical analysis to be used in the study. The normality of the data was determined by using Shapiro Wilk test. Between-group differences were analyzed using the Chi-square test with Fisher’s exact adjustment where appropriate for categorical variables and the t test for continuous variables. Statistical significance was expressed as p < 0.05. IBM SPSS v23.0 software was used to evaluate the data.

## Results

### Demographic and clinical characteristics of the patients

A total of 269 patients were followed. The average age of participants was 59.3 ± 15.6 years (range: 18–95 years) with 43.1% aged 65 and over. Males were 51.7% of patients enrolled. A significant portion (31.6%) of them had stage 4 chronic kidney disease (Table 1).

**Table 1:** Demographic and clinical characteristics of the patients (N = 269)

Characteristics	Values
Age, years	59.3 ± 15.6
<65, n (%)	153 (56.9)
≥65, n (%)	116 (43.1)
Male, n (%)	139 (51.7)
Female, n (%)	130 (48.3)
BMI, (kg/m^2^)	26.9 ± 6.1
Smoke, n (%)	
Yes	134 (49.8)
No	135 (50.2)
Alcohol, n (%)	
Yes	41 (15.2)
No	228 (84.8)
Allergy, n (%)	
Yes	48 (17.8)
No	221 (82.2)
Use of herbal, n (%)	
Yes	44 (16.4)
No	225 (83.6)
CKD grade, n (%)	
Common comorbidities, n (%)	
HT	181 (67.3)
DM	106 (39.4)
ASHD	60 (22.3)
G1	25 (9.3)
G2	29 (10.8)
G3a	27 (10.0)
G3b	50 (18.6)
G4	85 (31.6)
G5	52 (19.3)
Length of hospital stay, days	10.3 ± 6.9
Number of medications used on the first day	7.5 ± 3.3
Average number of medications used during hospitalization	8.8 ± 3.6

*ASHD* Atherosclerotic Heart Disease, *BMI* Body Mass Index, *CKD* Chronic Kidney Disease, *DM* Diabetes Mellitus, *G* Grade, *HT* Hypertension

The glomerular filtration rate was found to be 60 ml/min/1.73 m^2^ and above in 55 (20.4%) patients, and below 60 ml/min/1.73 m^2^ in 214 (79.6%) patients.

Patients (99.6%) had at least one comorbidity. The number of patients with three or more comorbidities was 194 (72.1%). The most common comorbidities observed in the patients were hypertension (67.3%), diabetes (39.4%), atherosclerotic heart disease (22.3%), dyslipidaemia (13.4%), coronary artery disease (11.2%) and heart failure (11.2%).

### Drug-related problems and recommendations

A total of 269 patients were admitted to the wards during study period. Clinical pharmacist reviewed all the patients, and 131 patients (48.7%) had at least one DRP. The average number of DRP per patient was 0.8 ± 1.0. A total of 205 DRPs were identified. Almost half (50.7%) of the 205 DRPs were treatment efficacy problems (i.e. pantoprazole without an indication of gastro-oesophageal reflux disease), 43.9% treatment safety, and 5.4% other issues (Table 2). A total of 213 DRP causes were identified. Drug selection (46.9%) (i.e. inappropriate drug according to guidelines/formulary, inappropriate drug (within guidelines but otherwise contra-indicated), no indication for drug, inappropriate combination of drugs, or drugs and herbal medications, or drugs and dietary supplements, inappropriate duplication of therapeutic group or active ingredient, no or incomplete drug treatment in spite of existing indication, too many drugs prescribed for indication) and dose selection (34.7%) were found to be the main causes of DRPs (Table 3). A total of 550 interventions were made for the resolution of 205 DRPs. If the solution to a problem concerned the nurse/patient, the same intervention was performed on both the nurse/patient and the physician. Therefore, the number of interventions was higher than the number of DRPs. Although most of the interventions (402/73.1%) were at the prescriber level, some were (123/22.4%) at the drug level. Most (188/91.7%) DRP interventions were accepted, but 10/4.9% were not, either by the patient or the physician (Table 2). A total of 205 DRPs were identified, and 147 (71.7%) DRPs were solved, while 4 (1.9%) DRPs were partially solved, 48 (23.4%) DRPs were unsolved, and 6 DRPs (2.9%) had unknown outcomes (Table 2).

**Table 2:** Types of drug-related problems and pharmacists’ interventions and outcomes according to the Pharmaceutical Care Network Europe DRP classification tool V8.03

Primary domain	PCNE Code	Number and Frequency (%)
**Problems**		N = 205
Treatment effectiveness	P1	104 (50,7)
No effect of drug treatment	P1.1	1 (0,5)
Effect of drug treatment not optimal	P1.2	69 (33,6)
Untreated symptoms or indication	P1.3	34 (16,6)
Treatment safety	P2	90 (43,9)
Adverse drug event (possibly) occurring	P2.1	90 (43,9)
Other	P3	11 (5,4)
Problem with cost-effectiveness of the treatment	P3.1	0 (0,0)
Unnecessary drug-treatment	P3.2	4 (2,0)
Unclear problem/complaint	P3.3	7 (3,4)
**Intervention**		**N = 550**
No intervention	I0	0 (0,0)
At prescriber level	I1	402 (73,1)
Prescriber informed only	I1.1	205 (37,3)
Prescriber asked for information	I1.2	2 (0,4)
Intervention proposed to prescriber	I1.3	195 (35,4)
At patient level	I2	25 (4,5)
Patient (drug) counselling	I2.1	25 (4,5)
At drug level	I3	123 (22,4)
Drug changed to …	I3.1	22 (4,0)
Dosage changed to …	I3.2	27 (4,9)
Formulation changed to …	I3.3	2 (0,4)
Instructions for use changed to …	I3.4	24 (4,4)
Drug paused or stopped	I3.5	30 (5,4)
Drug started	I3.6	18 (3,3)
Other intervention or activity	I4	0 (0,0)
Side effect reported to authorities	I4.2	0 (0,0)
**Implementation**		**N = 205**
Intervention accepted	A1	188 (91,7)
Intervention accepted and fully implemented	A1.1	147 (71,7)
Intervention accepted, partially implemented	A1.2	6 (2,9)
Intervention accepted but not implemented	A1.3	30 (14,6)
Intervention accepted, implementation unknown	A1.4	5 (2,4)
Intervention not accepted	A2	10 (4,9)
Intervention not accepted: no agreement	A2.2	9 (4,4)
Intervention not accepted: other reason (specify)	A2.3	1 (0,5)
Other	A3	7 (3,4)
Intervention not proposed	A3.2	7 (3,4)
**Outcome of intervention**		**N = 205**
Not known	O0	6 (2,9)
Problem status unknown	O0.1	6 (2,9)
Solved	O1	147 (71,7)
Problem totally solved	O1.1	147 (71,7)
Partially solved	O2	4 (1,9)
Problem partially solved	O2.1	4 (1,9)
Not solved	O3	48 (23,4)
Problem not solved, lack of cooperation of patient	O3.1	0 (0,0)
Problem not solved, lack of cooperation of prescriber	O3.2	13 (6,3)
Problem not solved; intervention not effective	O3.3	17 (8,3)
No need or possibility to solve problem	O3.4	18 (8,8)

*DRP* Drud Related Problem, *PCNE* Pharmacutical Care Network Europe

**Table 3:** Identified causes according to the PCNE DRP classification tool V8.03

Cause of the problem	PCNE Code	Number and Frequency (%)
Drug selection	C1	100 (46,9)
Inappropriate drug according to guidelines/formulary	C1.1	18 (8,4)
Inappropriate drug (within guidelines but otherwise contra-indicated)	C1.2	13 (6,1)
No indication for drug	C1.3	3 (1,4)
Inappropriate combination of drugs, or drugs and herbal medications, or drugs and dietary supplements	C1.4	31 (14,5)
No or incomplete drug treatment despite existing indication	C1.6	34 (16,0)
Too many drugs prescribed for indication	C1.7	1 (0,5)
Drug form	C2	2 (0,9)
Inappropriate drug form (for this patient)	C2.1	2 (0,9)
Dose selection	C3	74 (34,7)
Drug dose too low	C3.1	3 (1,4)
Drug dose too high	C3.2	28 (13,1)
Dosage regimen does not frequent enough	C3.3	6 (2,8)
Dosage regimen too frequent	C3.4	6 (2,8)
Dose timing instructions wrong, unclear or missing	C3.5	32 (15,0)
Treatment duration	C4	0 (0,0)
Dispensing	C5	8 (3.8)
Prescribed drug not available	C5.1	1 (0,5)
Necessary information not provided	C5.2	3 (1,4)
Wrong drug, strength or dosage advised (OTC)	C5.3	4 (1,9)
Drug use process	C6	5 (2,3)
Inappropriate timing of administration or dosing intervals	C6.1	4 (1,9)
Drug over-administered	C6.3	1 (0,5)
Patient related	C7	12 (5,6)
Patient takes food that interacts	C7.5	4 (1,9)
Patient stores drug inappropriately	C7.6	6 (2,8)
Patient administers/uses the drug in a wrong way	C7.8	1 (0,5)
Patient unable to use drug/form as directed	C7.9	1 (0,5)
Other	C8	12 (5,6)
No or inappropriate outcome monitoring (incl. TDM)	C8.1	6 (2,8)
Other cause	C8.2	6 (2,8)

*DRP* Drud Related Problem, *PCNE* Pharmacutical Care Network Europe

**Fig. 1:**
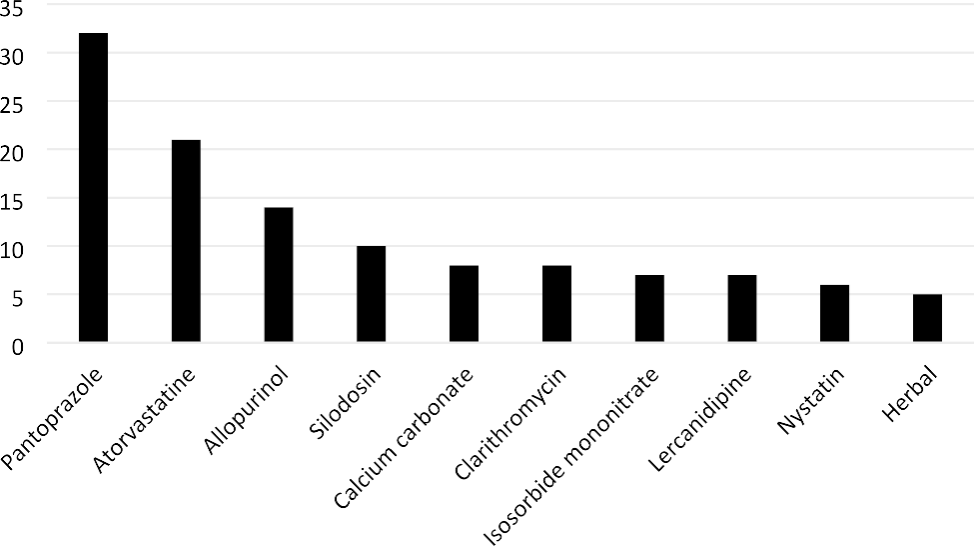
Drugs that most commonly cause DRPs

The most common drugs that cause DRPs are pantoprazole (15.6%), atorvastatin (10.2%), and allopurinol (6.8%) (Fig. 1). Pantoprazole was used without an indication in many patients or caused drug interactions. There is no DRP associated with acute interstitial nephritis. Furthermore, according to ATC (Anatomical Therapeutic Chemical) Classification codes the most common drug groups that lead to DRPs are drugs for gastrointestinal system (18.0%) and lipid metabolism (13.6%), and antibiotics (11.7%).

Approximately 49% of study patients (131) have at least one DRP. The prevalence of DRPs (55.0%) in female patients was significantly higher than male ones (p < 0.05). The hospital stays and the mean number of drugs used were significantly higher in the group with DRP than in the group without DRP (p < 0.05) (Table 4).

**Table 4:** Comparative analysis of patients with and without DRPs

	Total N = 269	With DRPs N = 131	Without DRPs N = 138	P value
Total	269	131 (48,7)	138 (51,3)	
Gender, n (%)				0,034^*^
Female	130 (48,4)	72 (55,0)	58 (42,0)	
Male	139 (51,6)	59 (45,0)	80 (58,0)	
Age (years)	59,3 ± 15,6	60,6 ± 15,7	58,1 ± 15,4	0,184
BMI (kg/m^2^)	26,9 ± 5,9	27,3 ± 6,2	26,6 ± 5,6	0,335
GFR (ml/min/1,73m^2^)	38,6 ± 29,9	36,6 ± 28,2	40,5 ± 31,4	0,490
CKD grade, n (%)				0,412
G 1–2	54 (20,1)	21 (7,8)	33 (12,3)	
G 3a-3b	77 (28,6)	41 (15,2)	36 (13,4)	
G 4	85 (31,5)	43 (15,9)	42 (15,6)	
G 5	52 (19,4)	26 (9,7)	26 (9,7)	
Length of hospital stay, (days)	10,3 ± 6,9	11,3 ± 7,7	9,3 ± 5,9	0,044^**^
Number of comorbidities	2,6 ± 1,5	2,7 ± 1,4	2,5 ± 1,6	0,460
Average number of medications used	8,8 ± 3,6	9,6 ± 3,6	8,1 ± 3,5	0,002^**^

*BMI* Body Mass Index, *CKD* Chronic Kidney Disease, *DRP* Drug Related Problem, *G* Grade, *GFR* Glomerular Filtration Rate

^*^ Statistically significant when compare with DRPs (Fisher’s exact test)

^**^ Statistically significant when compare with DRPs (Student t-test)

## Discussion

Clinical pharmacy services are crucial for CKD patients who require a complicated course of treatment [7]. With the use of this service, medical professionals such as physicians, nurses, dietitians, and clinical pharmacists work together as a multidisciplinary team to better manage CKD-related comorbid disorders [7]. In this study, we found that DRPs were present in 48.7% of the CKD patients. Although most (91.7%) DRP interventions were accepted by the physicians, and patient, 71.7% of DRPs were fully resolved. Treatment efficacy was found to be the main type of DRPs (56.2%).

In our study, 269 CKD patients were followed up. During the period, a total of 205 DRPs were found in 131 (48.7%) of 269 individuals. In a recently published meta-analysis research analyzing DRPs in hospitalized CKD patients, the prevalence of DRP was reported to range between 12 and 87% [8]. In studies conducted to investigate DRP, including CKD patients undergoing dialysis, Liu et al. [19] found DRP in 77% of patients, and Holm et al. [20] in 62% of patients. The fact that hemodialysis patients were excluded from our study may explain the higher prevalence of DRP in the two studies in comparison to our finding. In a French investigation of 103 patients with chronic renal disease, 394 DRPs were found in 93.2% of the patients. The comorbidity that arises with aging and the rising number of medications were associated with high risk of DRP in this study [21].

The average number of DRPs per patient was found to be 0.76 in the present study. This number varies between 0.36 and 3.8 in studies conducted in different countries [21–25]. Although each trial was conducted on CKD patients, the explanation for the disparate outcomes can be attributed to the fact that the number of medicines utilized varied depending on the patient subgroups covered.

In our study, the number of medicines was significantly higher in patients with DRP than without DRP (9.6 ± 3.6 and 8.1 ± 3.5, respectively) during their hospital stay (p < 0.05). Similarly, in a study conducted with 5217 chronic kidney patients in Germany, it was shown that each patient used an average of 8 drugs while in hospital [26].

This study found that 268 patients (99.6%) had at least one comorbidity. There were three or more comorbidities diagnosed in 72.1% of individuals. The most common comorbidities were found to be HT (67.3%), DM (39.4%), and atherosclerotic heart disease (22.3%). Similarly, HT, DM and anaemia are among the most common comorbidities in the study of Subeesh et al., and HT, DM, and CAD in the study of Rani et al. [22, 23]. However, there was no statistically significant difference in our study between individuals with (2.7 ± 1.4) and without (2.5 ± 1.6) DRP in terms of the presence of comorbidities (p > 0.05).

Clinical pharmacy services in hospital settings are beneficial in lowering treatment costs and delivering better treatment outcomes through the identification and resolution of DRP, according to substantial evidence in the literature [27–30]. Supporting these results, the length of hospital stays of patients with DRP (11.3 ± 7.7%) was found longer than patients without DRP (9.3 ± 7.7%) (p < 0.05). This finding implies that drug-related problems could have a negative impact on the efficacy and safety of the treatment as well as be a factor that raises the cost of care by extending the patient’s stay in the hospital.

In the current study most DRPs were related to either treatment effectiveness (50.7%) or treatment safety (43.9%). Likewise, Liu et al. [19] and Dvorackova et al. [24] found that the most frequent problems were treatment efficacy and drug selection as the cause in their analysis of PCNE in CKD patients. Drug interactions (21.8%) were identified by Njeri et al. [31] as the most frequent cause of DRPs. Drug interactions were found about 14% of the causes of DRPs in our study. Differently Subeesh et al. [22] found that drug interactions were the most common cause of DRP in CKD patients, including dialysis patients, at a high incidence (60%) according to PCNE assessment. Since dialysis patients taking more medications than other CKD patients, there may be a greater cause of drug interactions.

Gastrointestinal medications (18.0%), lipid metabolism drugs (13.6%), and antimicrobial drugs (11.7%) ranked 1–3 in the study’s analysis of the drug groups that are associated with DRPs. Compared to literature it is seen that antibiotics and medications for the gastrointestinal system are the two drug classes that most often associated with DRPs in individuals with chronic kidney disease [21, 24, 25].

In our study, over 92% of the suggestions given in relation to the issues found for DRP were accepted by physicians. This rate resembles results found in previous studies published in the CKD literature [19, 20, 32].

Despite the study’s high acceptance rate (91.7%), the rates of fully resolved DRP, partially resolved DRP, and unresolved DRP were 71.7%, 1.9%, and 23.4%, respectively. In a study conducted by Liu et al. [19], 76.1% of interventions were accepted, and 68.3% of DRPs were completely resolved. In a different study by Garedow et al. [25], 81.6% of interventions were accepted, and 79.8% of DRPs were fully resolved. Surprisingly, the percentage of resolved DRP in the study of Adibe et al. was very low. (7.9%) [33]. Although the rates of resolved DRPs were comparable, the cause of DRPs remained unresolved in our study may be related to logistics factors. The high acceptance rate of interventions and relatively high rates of resolved DRPs indicate the willingness of other healthcare providers to collaborate with clinical pharmacists. This is also an opportunity for optimizing care and practice of clinical pharmacy in Turkey.

### Limitations and strengths

This study has the following limitations. The lack of control group may attenuate the discrimination of the impact of clinical pharmacist from other factors that may lead to resolving DRP. We did not include dialysis patients as it is well studied in literature and our interest was in patients before they reach this stage to explore missed opportunities of care before reaching dialysis stage. Evaluation of long-term effect and impact on primary outcomes such as progression to an advanced stage of CKD and the need to be on dialysis was not done due to the limitations in logistics and financial support. Due to these reasons as well, the research was carried in one center. The results cannot be generalized to all CKD patients regarding DRPs because the study was only done in one center. We recommend further controlled multicenter studies, characterize to prevalence of DRP in CKD patients and the impact clinical pharmacist’s interventions. Another limitation is, although many factors related to the patient were evaluated, the effect of patient’s health literacy could not be evaluated in this study. Health literacy and patient’s compliance in patients can increase the number of problems related to drugs. Therefore, further studies are needed to examine these factors.

There are also strengths with the method used in this study. DRPs were evaluated in multidisciplinary healthcare teams, which may have resulted in a higher rate of interventions to the DRPs due to face-to-face conversations and interventions. Additionally, this is the first study that reports DRPs in nephrology ward by clinical pharmacists in Turkey.

## Conclusions

This is the first study that reports DRPs in nephrology ward by clinical pharmacists in Turkey, which proves that the clinical pharmacists play an active role in the drug safety of CKD patients. As a result, clinical pharmacy practices can assist the medically prescribed course of therapy by helping to avoid and address DRPs that may arise when treating CKD patients. In terms of the efficacy and safety of the treatment used in the Nephrology ward, it would be suitable to engage a clinical pharmacist alongside physicians, nurses, nutritionists, and other health professionals when reviewed collectively.

## Data Availability

The datasets generated and analysed during the current study are not publicly available because we are not allowed to share individual level data. However additional information about the data is available from the corresponding author on reasonable request.
